# Supplementary material for: Sickle Cell Hemoglobin Genotypes Affect Malaria Parasite Growth and Correlate with Exosomal miR-451a and let-7i-5p Levels
Source: Int J Mol Sci. 2023 Apr 19;24(8):7546. doi: 10.3390/ijms24087546 (PMC10141851; doi:10.3390/ijms24087546)
Supplement: Supplementary file 1 [file ijms-24-07546-s001.zip › ijms-2260105-supplementary.pdf]

Supplemental Figures:

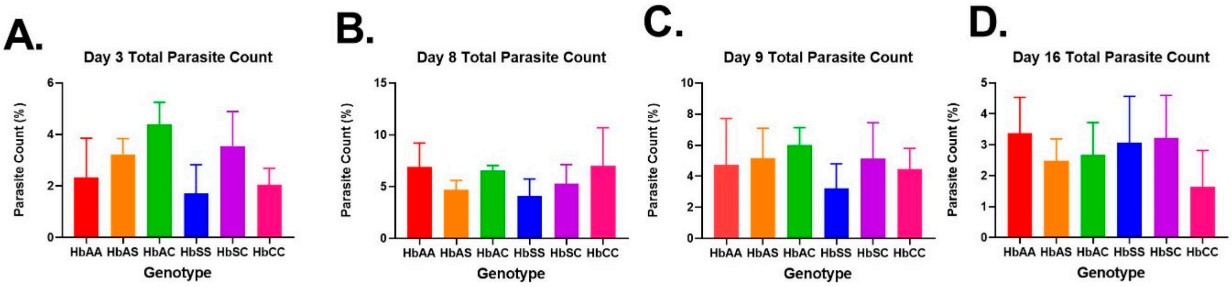

Supplemental Figure 1: Overall parasite counts between Hb genotypes for days 3, 8, 9 and 16. Red is for HbAA, orange for HbAS, green for HbAC, blue for HbSS, purple for HbSC, and pink for HbCC. Overall parasite count percentage and parasite phases rings, trophozoites and schizonts for each genotype for days 3, 8, 9, and 16 were compared between Hb genotypes using an ANOVA and a Tukey's multiple comparison test. **A-D**. Overall parasite count percentage for each genotype on days 3, 8, 9, and 16 respectively. No statistical significance was found between Hb genotypes.

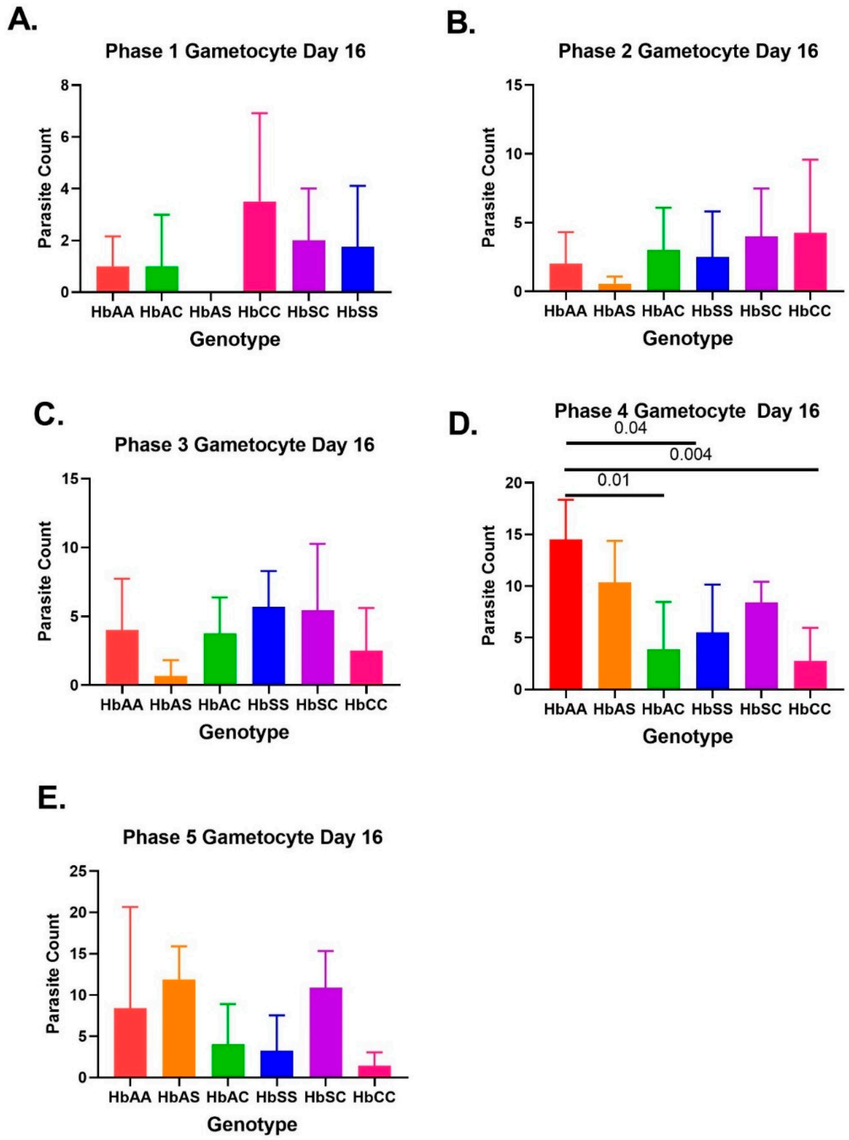

Supplemental Figure 2: Gametocyte phase 1-5 counts for each Hb genotype for day 16. Red is for HbAA, orange for HbAS, green for HbAC, blue for HbSS, purple for HbSC, and pink for HbCC. Gametocytes have five distant physiological phases. An ANOVA and Tukey's multiple comparison test was used to compare parasite count for each phase between Hb genotypes on day 16. **A.** There was no significant difference of number of phase 1 gametocytes. **B.** There was no significant difference of number of phase 2 gametocytes. **C.** There was no significant difference of number of phase 3 gametocytes. **D.** HbAA had significantly more phase 4 gametocytes than HbAC (P=0.01), HbSS (P=0.04), and HbCC (P=0.004). **E**. There was no significant difference of number of phase 5 gametocytes.

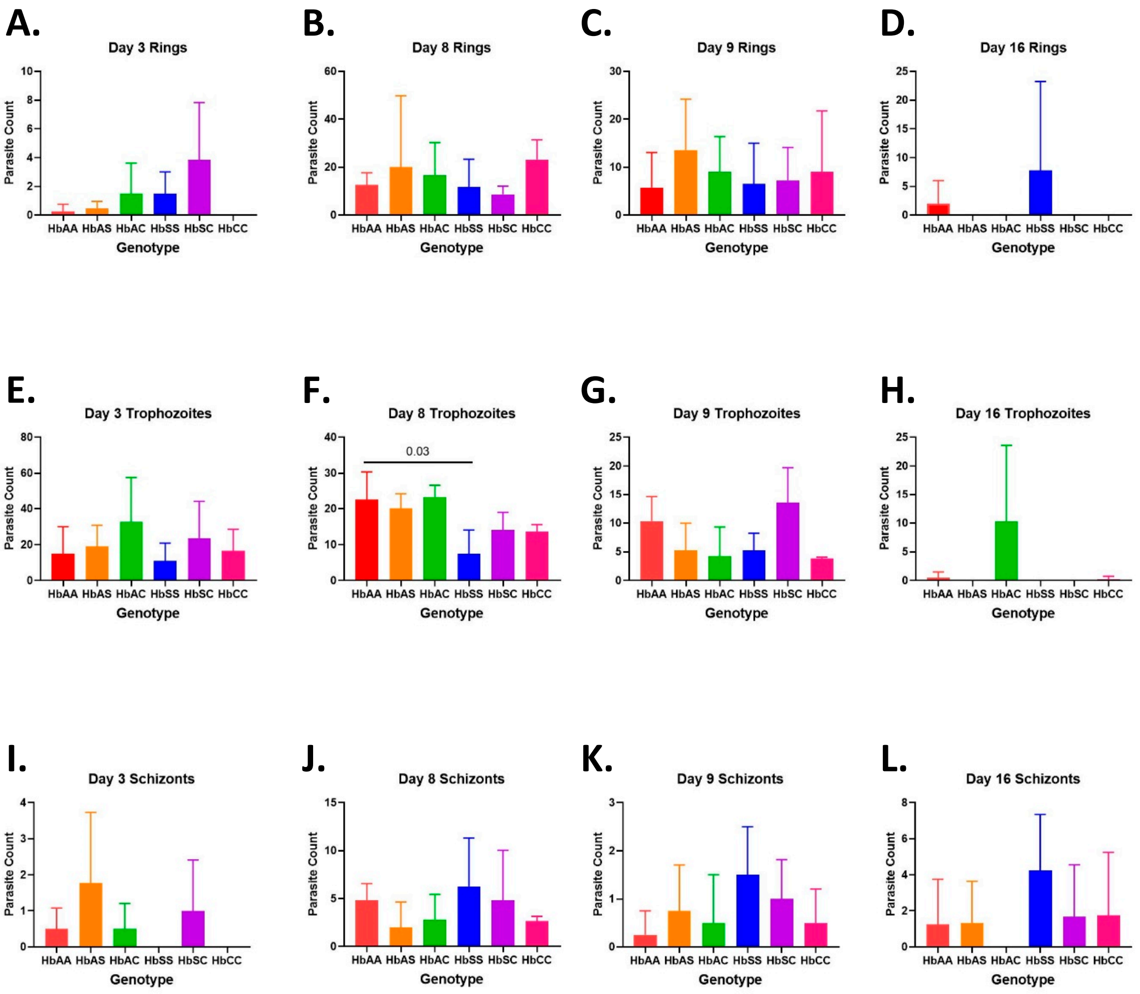

Supplemental Figure 3: Counts of different parasite phases between Hb genotypes for days 3, 8, 9 and 16. A-D. Ring counts for each genotype for days 3, 8, 9, and 16, respectively. No statistical significance was found**. E-H.** Trophozoite counts for each genotype for days 3, 8, 9, and 16, respectively. There was only one significant difference between Hb genotypes on day 8 where HbAA had more trophozoites (P=0.03) compared to HbSS. **I-L**. Schizonts counts for each genotype for days 3, 8, 9, and 16, respectively. No statistical significance was found with n=4 per genotype.

Table 1. Patient hematological characteristics table

CHARACTERISTICS (MEAN±SD)	HbAA N= 4	HbAS N=4	HbAC N=4	HbSS N=4	HbSC N=4	HbCC N=4
Age (years)	30.8 ±8.5	25 ± 9.2	32.5 ± 10.6	26.3 ± 9.5	35.3 ± 0.5	32.3 ± 4.5
Sex (male)	2	2	1	2	2	3
Bloody Type:						
AB+	0	0	0	0	1	0
A+	1	1	0	1	2	0
B+	2	1	2	1	0	2
O+	1	2	2	2	1	2
WBC ($x10^3/mm^3$)	5.4 ± 0.8	5.9 ± 0.5	5.8 ± 1.7	12.1 ± 6.7	8.5 ± 5.4	5.6 ± 2.6
RBC ($x10^6$/ µl)	4.6 ± 0.4	4.7 ±0.5	4.5 ± 0.6	3.3 ± 1.1	4.4 ± 0.4	3.7 ± 1
Hb (g/dl)	14.2 ± 2.6	14.2 ± 1.8	13.4 ± 1.6	11.9 ± 2.7	11.2 ± 1.3	10.2 ± 1.8
Platelets ($x10^3$/ µl)	241.8 ± 19.5	221.3 ± 33.9	284.8 ± 144.6	348.3 ± 174	220.5 ± 72.1	174 ± 76.4

Table 2: Comparison of exosomal miR-451a and let-7i-5p for days 3, 8, 9, and 16 for each genotype in RBC vs iRBCs.

Day	Genotype Comparison	miR-451a	Let-7i-5p
**3**	HbAA- vs HbAA+	NS	NS
	HbAS- vs HbAS+	NS	NS
	HbAC- vs HbAC+	NS	NS
	HbSC- vs HbSC+	NS	NS
	HbSS- vs HbSS+	NS	NS
	HbCC- vs HbCC+	NS	NS
**8**	HbAA- vs HbAA+	NS	NS
	HbAS- vs HbAS+	NS	NS
	HbAC- vs HbAC+	NS	**0.04**
	HbSC- vs HbSC+	NS	NS
	HbSS- vs HbSS+	**0.03**	NS
	HbCC- vs HbCC+	NS	NS
**9**	HbAA- vs HbAA+	NS	NS
	HbAS- vs HbAS+	NS	NS
	HbAC- vs HbAC+	NS	NS
	HbSC- vs HbSC+	NS	NS
	HbSS- vs HbSS+	NS	NS
	HbCC- vs HbCC+	NS	NS
**16**	HbAA- vs HbAA+	NS	NS
	HbAS- vs HbAS+	NS	NS
	HbAC- vs HbAC+	NS	NS
	HbSC- vs HbSC+	NS	NS
	HbSS- vs HbSS+	NS	NS
	HbCC- vs HbCC+	NS	NS

Table 2: -= malaria negative, +=malaria positive, NS= not significant, P>0.05. All comparisons were analyzed using an unpaired t-test.
